# Possible Attenuation of Early Weight Loss During Tirzepatide Therapy in a Patient Treated With Levosulpiride: A Case Report and Mechanistic Hypothesis

**DOI:** 10.7759/cureus.111508

**Published:** 2026-06-25

**Authors:** Augusto Carducci, Pierfrancesco Di Matteo, Claudio Ferri, Davide Grassi

**Affiliations:** 1 Internal Medicine, Università degli Studi dell'Aquila, L'Aquila, ITA

**Keywords:** glp-1 receptor agonists, levosulpiride, tirzepatide, type 2 diabetes, weight loss and obesity

## Abstract

Tirzepatide, a dual glucose-dependent insulinotropic polypeptide (GIP) and glucagon-like peptide-1 (GLP-1) receptor agonist, induces significant weight loss and glycemic improvement. Dopaminergic pathways are critically involved in appetite regulation and reward processing, and the pharmacological modulation of dopamine signaling may influence metabolic responses to incretin-based therapies. We report the case of a 54-year-old woman with obesity and newly diagnosed type 2 diabetes mellitus (T2DM) who initiated tirzepatide therapy while receiving levosulpiride for functional dyspepsia. During the co-administration of tirzepatide 5 mg weekly and levosulpiride, only modest weight reduction was observed, followed by a plateau. After the discontinuation of levosulpiride and the titration of tirzepatide to 7.5 and 10 mg weekly, a marked and progressive reduction in body weight occurred, with an overall loss of 11 kg from baseline. Glycemic control improved substantially (glycated hemoglobin {HbA1c}: 5.0%), and no episode of pancreatitis occurred despite asymptomatic lipase elevation. This case raises the hypothesis of a potential pharmacodynamic interaction between dopaminergic antagonism and incretin receptor agonism, possibly mediated by opposing effects on gastric motility and central reward pathways. Although causality cannot be established, clinicians should consider concomitant neuroactive medications in cases of suboptimal early weight response to dual incretin therapy.

## Introduction

Tirzepatide is a novel dual agonist of glucose-dependent insulinotropic polypeptide (GIP) and glucagon-like peptide-1 (GLP-1) receptors that has demonstrated substantial efficacy in improving glycemic control and inducing clinically meaningful weight loss in patients with type 2 diabetes mellitus (T2DM) and obesity. In large, global phase 3 trials encompassing the SURPASS and SURMOUNT programs, which evaluated once-weekly subcutaneous tirzepatide across a maintenance dose range of 5 mg to 15 mg, this dual GIP/GLP-1 receptor (GLP-1R) agonist demonstrated profound efficacy. Over treatment durations of 40-72 weeks, tirzepatide produced dose-dependent reductions in glycated hemoglobin (HbA1c) of up to 2.4%-2.7% and body weight reductions of 12.4-15.6 kg (13.1%-15.7%) in patients with type 2 diabetes. In head-to-head comparisons, tirzepatide significantly outperformed selective GLP-1 receptor agonists and basal insulin therapy; for instance, in the 40-week SURPASS-2 trial, the 15 mg dose achieved an HbA1c decrease of 0.44% greater and a weight loss of 6.2 kg greater than semaglutide 1 mg. Similarly, in the 52-week SURPASS-4 trial against titrated insulin glargine, tirzepatide 15 mg achieved an additional 1.14% reduction in HbA1c and an average weight difference of 9.8 kg while avoiding the weight gain typically associated with insulin escalation [1-4].

The metabolic effects of tirzepatide result from a combination of peripheral and central mechanisms. Beyond enhancing glucose-dependent insulin secretion and reducing glucagon levels, GLP-1 receptor activation modulates hypothalamic pathways involved in satiety and interacts with mesolimbic dopaminergic circuits that regulate food reward and motivational behavior [5,6]. Dual GIP/GLP-1 receptor activation may further amplify these effects through additive intracellular signaling at the level of pancreatic β-cells and central appetite-regulating networks [7].

Dopamine signaling plays a fundamental role in feeding behavior, reward processing, and energy homeostasis. The pharmacological antagonism of dopamine D2 receptors has been associated with metabolic alterations, hyperprolactinemia, insulin resistance, and weight gain, particularly in patients treated with antipsychotic drugs [8,9]. Levosulpiride, a selective D2/D3 receptor antagonist widely prescribed as a prokinetic agent, exerts central dopaminergic effects and increases prolactin secretion through the inhibition of the tuberoinfundibular pathway [10].

We report a case of a patient with obesity and newly diagnosed T2DM in whom early weight loss during tirzepatide therapy appeared attenuated during concomitant levosulpiride administration, followed by marked weight reduction after its discontinuation.

## Case presentation

A 54-year-old woman (height: 164 cm) with a history of obesity, metabolic syndrome, mixed dyslipidemia, and euthyroid autoimmune thyroiditis (stable thyroid function, no levothyroxine replacement therapy) presented with progression from prediabetes to T2DM in June 2025. Family history was significant for diabetes mellitus in both parents and premature cardiovascular disease in first-degree relatives. She had never smoked and reported low alcohol intake (approximately 13 g/week). During the observation period, the patient's physical activity was initially absent in 2024 and became light during 2025, increasing from highly sedentary (<2,500 steps/day) to lightly active (~5,000 steps/day).

Baseline evaluation showed a body weight of 82 kg (BMI: 30.5 kg/m²), a glycated hemoglobin of 5.9% in May 2024 and 5.4% in December 2024, a low-density lipoprotein (LDL) cholesterol of 152 mg/dL, triglycerides of 233 mg/dL, and a fasting insulin of 17.2 μU/mL, consistent with insulin resistance. In June 2025, an oral glucose tolerance test (OGTT) revealed a marked early glycemic spike, with a 30-minute plasma glucose value reaching 220 mg/dL, highlighting significant postprandial glucose intolerance. To rigorously establish the diagnosis of type 2 diabetes mellitus (T2DM) according to standard international guidelines, fasting plasma glucose was subsequently evaluated, confirming diabetes with a value of 127 mg/dL in two separate, independent determinations.

Her medical history included gastroesophageal reflux disease (GERD) with grade B esophagitis and hiatal hernia, hepatic steatosis (abdominal ultrasound evidence of fatty liver), chronic gastritis, frequent ventricular extrasystoles, mild mitral regurgitation, lumbar disc disease, and diverticulosis.

On March 4, 2025, the patient was prescribed levosulpiride oral drops (25 mg/mL) at a dosage of eight drops three times daily (equivalent to approximately 8 mg per dose, 24 mg/day total) for the management of gastroesophageal reflux disease (GERD). 

Tirzepatide therapy was started on June 25, 2025, at a dose of 5 mg weekly. The same dose was administered again on July 21, 2025. On August 18, 2025, the dose was increased to 7.5 mg weekly and on September 15, 2025, to 10 mg weekly, which was subsequently maintained. Concomitant therapy added on December 23, 2025, included dapagliflozin 10 mg daily and ezetimibe 10 mg daily.

During the period of co-administration of tirzepatide 5 mg and levosulpiride (June 25 to August 18, 2025), body weight decreased from 82 kg to 79 kg and subsequently plateaued at 79 kg until mid-September. Levosulpiride was discontinued on August 18, 2025, coinciding with the escalation of tirzepatide to 7.5 mg.

Following the discontinuation of levosulpiride and the further titration of tirzepatide to 10 mg, progressive weight loss was observed. By December 23, 2025, body weight had decreased to 71 kg (BMI: 26.4 kg/m²), corresponding to an overall reduction of 11 kg from baseline and 8 kg after the suspension of levosulpiride.

**Figure 1:**
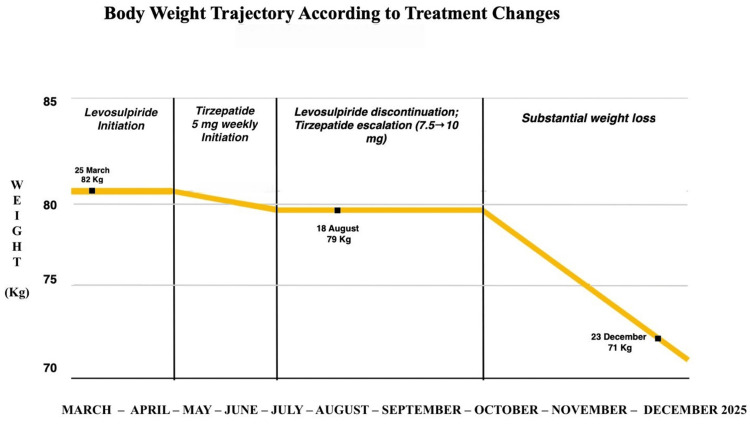
Trend of Body Weight Over Time With and Without Levosulpiride The temporal relationship between tirzepatide dose escalation, levosulpiride discontinuation, and body weight changes. The x-axis represents time (March-December 2025), while the y-axis represents body weight (kg). Initial therapy with tirzepatide 5 mg weekly during concomitant levosulpiride administration was associated with modest weight reduction (82 kg to 79 kg), followed by a plateau phase. After the discontinuation of levosulpiride and the escalation of tirzepatide to 7.5 mg and subsequently 10 mg weekly, progressive and sustained weight loss occurred, reaching 71 kg at follow-up. Vertical markers indicate key therapeutic changes (created by the author using Microsoft PowerPoint (Microsoft Corp., Redmond, WA)

Glycemic control improved markedly, with HbA1c values of 5.4% in October and November 2025 and 5.0% in December 2025. The estimated glomerular filtration rate, transiently reduced to 85 mL/minute/1.73 m² in October and November 2025, improved to 103 mL/minute/1.73 m² in December 2025 (Figure 1). Liver function markers remained stable and well within clinically acceptable variations throughout the entire observation period, showing no pathological shifts from baseline to the final evaluation (baseline: aspartate aminotransferase {AST}, 23 U/L; alanine aminotransferase {ALT}, 38 U/L; gamma-glutamyl transferase {GGT}, 40 U/L; December 2025: AST, 20 U/L; ALT, 39 U/L; GGT, 57 U/L), consistent with known hepatic steatosis. During the early titration phase, a transient, asymptomatic elevation in serum lipase was observed, rising from a baseline of 58 U/L to a peak of 94 U/L (approximately 1.5 times the upper limit of normal {ULN} of 60 U/L). This laboratory finding occurred in the absolute absence of abdominal pain, nausea, or clinical features suggestive of acute pancreatitis, while serum amylase consistently remained within normal limits. Serial clinical examinations and follow-up abdominal imaging confirmed entirely normal pancreatic morphology, ruling out subclinical pancreatic disease and supporting the continuation of the tirzepatide titration protocol.

## Discussion

This case highlights a temporal association between concomitant levosulpiride therapy and the attenuation of early weight loss during the initiation of tirzepatide. Although a causal relationship cannot be established, several pharmacological mechanisms may plausibly explain this observation.

Tirzepatide exerts weight-reducing effects through multiple complementary pathways. At the receptor level, it activates both GLP-1R and GIPR, favoring cyclic adenosine monophosphate (cAMP)-mediated intracellular signaling with reduced β-arrestin recruitment, potentially prolonging metabolic effects [11,12]. Clinically, GLP-1 receptor activation delays gastric emptying, enhances satiety signaling, stimulates proopiomelanocortin neurons in the hypothalamus, and modulates dopaminergic reward circuits in the ventral tegmental area and nucleus accumbens [5,6]. Early weight reduction during the first weeks of therapy is partly attributed to delayed gastric emptying and reduced caloric intake.

Levosulpiride, by contrast, antagonizes dopamine D2 receptors and acts as a partial 5-HT4 receptor agonist, accelerating gastric emptying and enhancing gastrointestinal motility [10,13]. This represents a pharmacodynamic opposition to the gastric-slowing effect of tirzepatide, particularly relevant during the initial low-dose phase when gastric emptying delay is most pronounced.

Beyond gastrointestinal effects, dopaminergic pathways play a central role in feeding motivation and metabolic regulation. Chronic D2 receptor antagonism has been associated with hyperprolactinemia, insulin resistance, and weight gain in other clinical contexts [8,9]. Increased prolactin levels secondary to D2 blockade may contribute to metabolic alterations.

A notable limitation of the present report is the absence of longitudinal serum prolactin measurements during concurrent levosulpiride therapy and after its withdrawal. While D2 receptor antagonism is firmly established to induce hyperprolactinemia, which can fundamentally alter metabolic homeostasis, suppress lipolysis, and promote fluid retention, the specific involvement of the tuberoinfundibular prolactin pathway remains a theoretical, modulatory mechanism in this patient due to the lack of direct biochemical confirmation.

Consequently, the observed clinical pattern must not be overinterpreted as a purely prolactin-driven phenomenon. Instead, it should be conceptualized as a complex pharmacodynamic intersection where dopaminergic blockade may have centrally or peripherally blunted the early anorectic, metabolic, or gastrointestinal pathways of dual incretin therapy. This limitation underscores the need for future studies to systematically include neuroendocrine biomarkers while reinforcing the practical clinical need to proactively review concomitant neuroactive or prokinetic medications in patients exhibiting a suboptimal early weight response to GIP/GLP-1 receptor agonists.

In the present case, the discontinuation of levosulpiride was followed by the restoration of progressive weight loss in parallel with tirzepatide dose escalation. While dose titration represents a confounding factor, the temporal relationship suggests that dopaminergic antagonism may have attenuated early anorectic or gastrointestinal effects of dual incretin therapy.

Given the expanding use of GLP-1 receptor agonists and dual GIP/GLP-1 agonists in diabetes and obesity management, a review of concomitant neuroactive medications may be advisable in patients with suboptimal early weight response.

## Conclusions

In this patient with obesity and newly diagnosed T2DM, concomitant levosulpiride therapy was temporally associated with reduced early weight loss during tirzepatide initiation. After the discontinuation of the dopamine D2 antagonist and the titration of tirzepatide, marked and progressive weight reduction was observed. This overlapping timeline limits our ability to definitively isolate the independent metabolic effects of levosulpiride withdrawal from the expected, dose-dependent efficacy of the dual GIP/GLP-1 receptor agonist.

Although causality cannot be inferred from a single case, mechanistic plausibility is supported by receptor pharmacology, opposing effects on gastric motility, dopaminergic-incretin cross-talk, and endocrine considerations. Prospective studies are needed to clarify the clinical relevance of this potential interaction. Future research should ideally isolate these confounding variables by evaluating the introduction or withdrawal of dopamine antagonists in patient cohorts maintained on stable, unchanged maintenance doses of incretin therapies while systematically tracking robust neuroendocrine biomarkers, specifically serum prolactin, and utilizing validated, patient-reported satiety scores to unravel the precise central and peripheral mechanisms involved.

## References

[1] Frías JP, Davies MJ, Rosenstock J (2021). Tirzepatide versus semaglutide once weekly in patients with type 2 diabetes. N Engl J Med.

[2] Del Prato S, Kahn SE, Pavo I (2021). Tirzepatide versus insulin glargine in type 2 diabetes and increased cardiovascular risk (SURPASS-4): a randomised, open-label, parallel-group, multicentre, phase 3 trial. Lancet.

[3] Jastreboff AM, Aronne LJ, Ahmad NN (2022). Tirzepatide once weekly for the treatment of obesity. N Engl J Med.

[4] Sinha R, Papamargaritis D, Sargeant JA, Davies MJ (2023). Efficacy and safety of tirzepatide in type 2 diabetes and obesity management. J Obes Metab Syndr.

[5] Holst JJ (2007). The physiology of glucagon-like peptide 1. Physiol Rev.

[6] Skibicka KP (2013). The central GLP-1: implications for food and drug reward. Front Neurosci.

[7] Finan B, Ma T, Ottaway N (2013). Unimolecular dual incretins maximize metabolic benefits in rodents, monkeys, and humans. Sci Transl Med.

[8] Reynolds GP, Kirk SL (2010). Metabolic side effects of antipsychotic drug treatment--pharmacological mechanisms. Pharmacol Ther.

[9] Peveler RC, Branford D, Citrome L (2008). Antipsychotics and hyperprolactinaemia: clinical recommendations. J Psychopharmacol.

[10] Tonini M, Cipollina L, Poluzzi E, Crema F, Corazza GR, De Ponti F (2004). Review article: clinical implications of enteric and central D2 receptor blockade by antidopaminergic gastrointestinal prokinetics. Aliment Pharmacol Ther.

[11] Willard FS, Douros JD, Gabe MB (2020). Tirzepatide is an imbalanced and biased dual GIP and GLP-1 receptor agonist. JCI Insight.

[12] Sun B, Willard FS, Feng D (2022). Structural determinants of dual incretin receptor agonism by tirzepatide. Proc Natl Acad Sci U S A.

[13] García-Tornadú I, Ornstein AM, Chamson-Reig A (2010). Disruption of the dopamine d2 receptor impairs insulin secretion and causes glucose intolerance. Endocrinology.

